# Femtosecond laser preparation of resin embedded samples for correlative microscopy workflows in life sciences

**DOI:** 10.1063/5.0142405

**Published:** 2023-04-03

**Authors:** Carles Bosch, Joerg Lindenau, Alexandra Pacureanu, Christopher J. Peddie, Marta Majkut, Andrew C. Douglas, Raffaella Carzaniga, Alexander Rack, Lucy Collinson, Andreas T. Schaefer, Heiko Stegmann

**Affiliations:** 1 Sensory Circuits and Neurotechnology Laboratory, The Francis Crick Institute, London, United Kingdom; 2 Carl Zeiss Microscopy GmbH, Oberkochen, Germany; 3 ESRF, The European Synchrotron, Grenoble, France; 4 Electron Microscopy STP, The Francis Crick Institute, London, United Kingdom; 5 Department of Neuroscience, Physiology and Pharmacology, University College London, London, United Kingdom

## Abstract

Correlative multimodal imaging is a useful approach to investigate complex structural relations in life sciences across multiple scales. For these experiments, sample preparation workflows that are compatible with multiple imaging techniques must be established. In one such implementation, a fluorescently labeled region of interest in a biological soft tissue sample can be imaged with light microscopy before staining the specimen with heavy metals, enabling follow-up higher resolution structural imaging at the targeted location, bringing context where it is required. Alternatively, or in addition to fluorescence imaging, other microscopy methods, such as synchrotron x-ray computed tomography with propagation-based phase contrast or serial blockface scanning electron microscopy, might also be applied. When combining imaging techniques across scales, it is common that a volumetric region of interest (ROI) needs to be carved from the total sample volume before high resolution imaging with a subsequent technique can be performed. In these situations, the overall success of the correlative workflow depends on the precise targeting of the ROI and the trimming of the sample down to a suitable dimension and geometry for downstream imaging. Here, we showcase the utility of a femtosecond laser (fs laser) device to prepare microscopic samples (1) of an optimized geometry for synchrotron x-ray tomography as well as (2) for volume electron microscopy applications and compatible with correlative multimodal imaging workflows that link both imaging modalities.

**FIG. 1.**
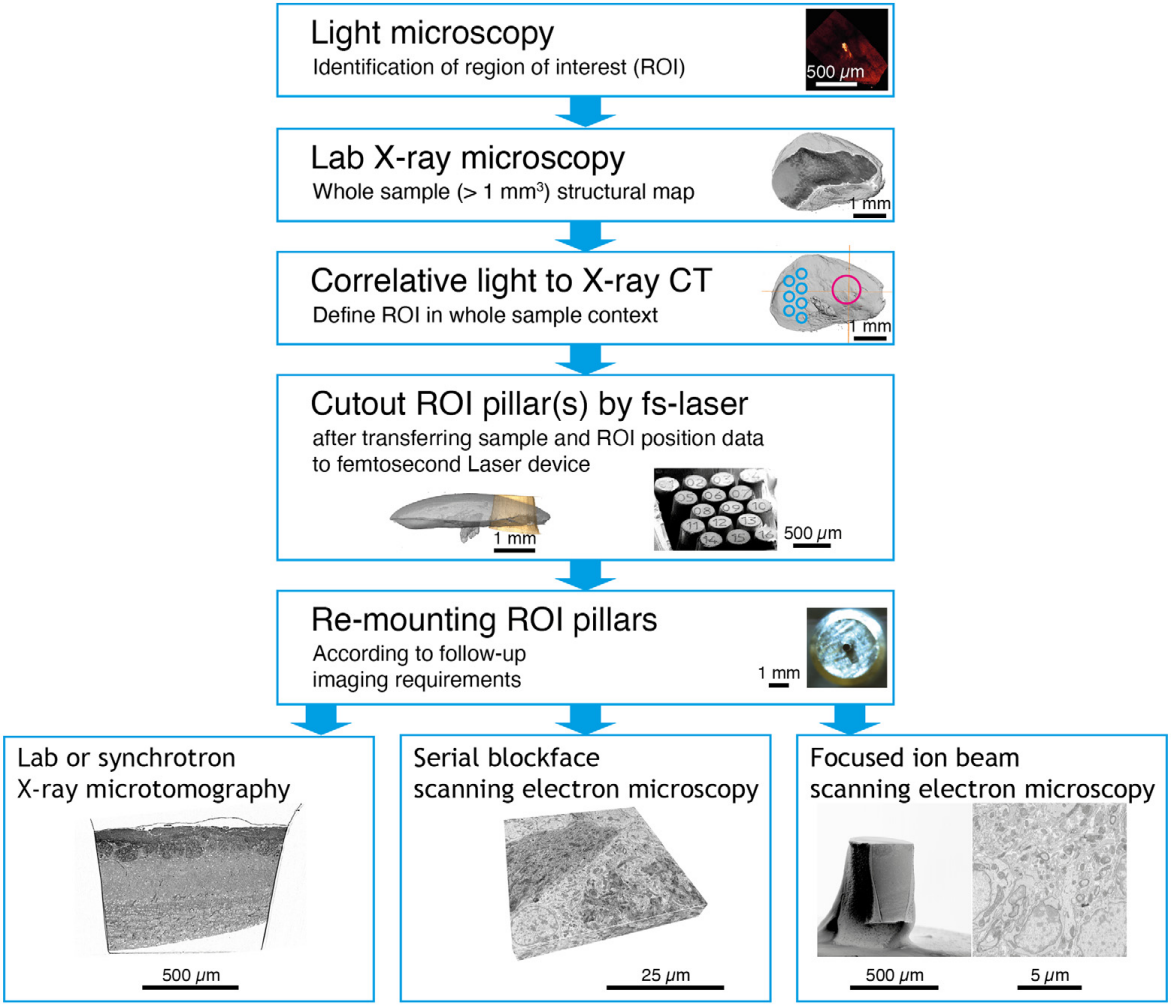
Correlative microscopy workflows incorporating samples processed using a femtosecond laser.

A femtosecond laser (fs laser) is an ultra-short pulse laser with pulse lengths typically ranging between a few ten and a few hundred femtoseconds.^1–4^ A fs laser allows rapid material removal to access targeted regions within a larger sample. Here, we used a fs laser with a wavelength of 515 nm, pulse length <350 fs, adjustable pulse repetition rate of 1 kHz to 1 MHz, average power 10 W at 1 MHz (max. pulse energy 10 *μ*J at 1 MHz), and focus spot diameter of <15 *μ*m. The laser was integrated in a FIB-SEM system (Zeiss Crossbeam 350) allowing for a rapid sample and laser target coordinate transfer between the laser processing chamber and the main chamber of the instrument.^1,2^ The fs laser was positioned in a separate chamber attached directly to the main Crossbeam chamber via an airlock. This design allows the ablation of large sample volumes without contaminating detectors or other sensitive components of the main chamber. Since fs lasers provide athermal material ablation,^4,5^ the laser affected zone (LAZ) is minimized, extending less than 0.5 *μ*m into a laser cut cross section. The narrow LAZ enables the targeting of milling boundaries with an accuracy in the range of *μ*m. The geometry of the carved specimen can be arbitrary defined by the operator, including curved surfaces, making it trivial to generate samples in the shape of cylindrical pillars, a feature also at reach when milling the samples using lathe systems^6^ but not when using microtome-based approaches. The fs lasers enable high ablation rates of up to several 10^6^ *μ*m^3^/s (Fig. S1 of the supplementary material), depending on the sample material and the laser settings used, and are compatible with heavy metal-stained, resin-embedded biological soft tissues.^7,8^

Altogether, these characteristics make the fs laser a suitable tool to extract targeted volumes of interest from a larger sample, the size, coordinates, and geometry of which were previously defined with compatible imaging techniques. Moreover, since the milling operation only requires access to one face of the resin block, multiple milling operations can be performed on a single specimen, enabling the extraction of tens of samples from neighboring regions in the same specimen.

This versatility places fs lasers in a potentially highly useful niche, enabling the generation of multiple independent specimens containing targeted volumes of interest from sub-cubic millimeter to several cubic millimeters in size and with optimized geometry for follow-up imaging techniques (Fig. 1). In these specimens, the histological detail can be efficiently retrieved at multiple scales in 3D using several distinct imaging technologies—from synchrotron x-ray computed tomography with propagation-based phase contrast^9^ or nano-holographic tomography^10^ to serial blockface scanning electron microscopy (SBF-SEM)^11–13^ and ultimately focused ion beam scanning electron microscopy (FIB-SEM).^14,15^

The use of fs lasers for targeted sample preparation in materials science is a well-established method and has been applied in a variety of different studies.^1,16^ Utilization of the fs laser for sample processing in life sciences provides a tool for targeted trimming of soft biological tissue that can complement other available mechanical tools in workflows that are highly dependent on precise specimen trimming, such as ultramicrotomy^17^ or lathe implementations.^6^ To interrogate the ultrastructure at nanometer scale, tissue specimens of dimensions reaching several cubic millimeters need to be contrasted with heavy metals and embedded in resin.^18–25^ Studying rare, localized structures with sub-micrometer detail requires a highly accurate preparation of those volumes of interest to be imaged and analyzed.^23,26–28^ Both preparation and imaging techniques will often impose sample size restrictions. Altogether, experimental targeting and sample size restrictions will define how the stained specimen will have to be trimmed, to ultimately match the downstream imaging modality. Finally, extracting multiple targeted samples from a single stained specimen is very challenging with conventional techniques, and therefore, at this point, it is more common to aim to prepare only one final sample per resin-embedded specimen. This, however, not only limits the usability of potentially highly precious specimens but also prevents the combined analysis of spatially distant ultrastructural features.

When looking at x-ray tomography of resin-embedded biological samples, additional challenges and limitations apply for sample preparation. In this case, for optimal results, samples are preferred to be cylindrical in geometry to reduce missing edge artifacts,^29^ with specific imaging techniques and specimens imposing upper bounds on the optimal diameter range.^9^ The femtosecond laser trimming is a technique uniquely suited to prepare multiple cylindrical samples of specific diameters (200–1000 *μ*m), all originating from a single specimen (Fig. S1 of the supplementary material).

In order to assess the feasibility of a fs laser for sample preparation of biological tissue, we investigated two use cases, namely, preparation for (1) targeted x-ray computed tomography and (2) multiple samples for serial blockface or focused ion beam volume EM acquisition.

We first assessed the capability of fs lasers to ablate resin (specifically epoxy resin EMbed 812 as typically used for embedding samples for electron microscopy, in particular, for volume EM methods where physical sectioning is involved). Laser power, pulse frequency, scan speed, and scan strategy were varied to identify settings that provided cleanly cut pillars with minimum surface roughness. With the optimum laser settings found (see methods), arrays of pillars of 450–830 *μ*m height and a pillar diameter down to 250 *μ*m (measured at the tip) were obtained.

In order to define a specific target region, we employed *ex vivo* two-photon imaging in a mouse line where one specific biological structure, an olfactory bulb glomerulus^9,30^ was genetically labeled [Fig. 2(a)]. In this case, two-photon imaging was performed after dissection of the brain, preparation of a 600 *μ*m horizontal brain slice, and GA/PFA fixation (see methods). Subsequently and in preparation for high-resolution x-ray tomography and EM, we stained the tissue with heavy metals (see methods and Ref. 23). Using lab x-ray tomography as described before^9^ allowed us to identify the very region previously imaged using two-photon microscopy [Figs. 2(b) and 2(c)]. We then transferred the fixed, stained, and embedded brain slice into an SEM with a coupled fs laser^1,2^ (for the combined FIB-SEM system, see methods, but the volume EM capabilities of this system were not used in this study). The volume containing the region of interest was delineated [magenta, Fig. 2(d)] as well as several smaller, non-targeted regions (blue). Targeting and separation are shown by secondary electron imaging in Figs. 2(e) and 2(f). These cylindrical pillars could then be further imaged using multiple techniques, such as EM or synchrotron x-ray computed tomography with propagation-based phase contrast (SXRT).^9^ Overall, the milling time was 15–24 min to extract ∼15 pillars from a specimen. This demonstrates the possibility of using fs lasers to define features and extract ROIs in fixed, metal stained, resin-embedded biological tissue. To apply this for the preparation for synchrotron x-ray tomography, we sought to obtain near-cylindrical samples with smooth surfaces as these result in the highest resolution and most-efficient data acquisition for tomography. Figures 2(g) and 2(h) show such an example where again a region containing a genetically labeled glomerulus is marked on a lab x-ray CT (LXRT) tomogram and subsequently milled on the above-mentioned fs-SEM system. The resulting trimmed volume can be readily imaged at a parallel beam SXRT beamline and it contains the target region of interest, resulting in high-resolution tomograms where individual dendrites of experimentally relevant neuronal circuits are readily visible as expected [Figs. 2(h) and 2(i)]. Thus, fs lasers, targeted to specific sample regions based on LXRT coordinates, can be used to reliably prepare samples optimized for subsequent x-ray tomography.

**FIG. 2.**
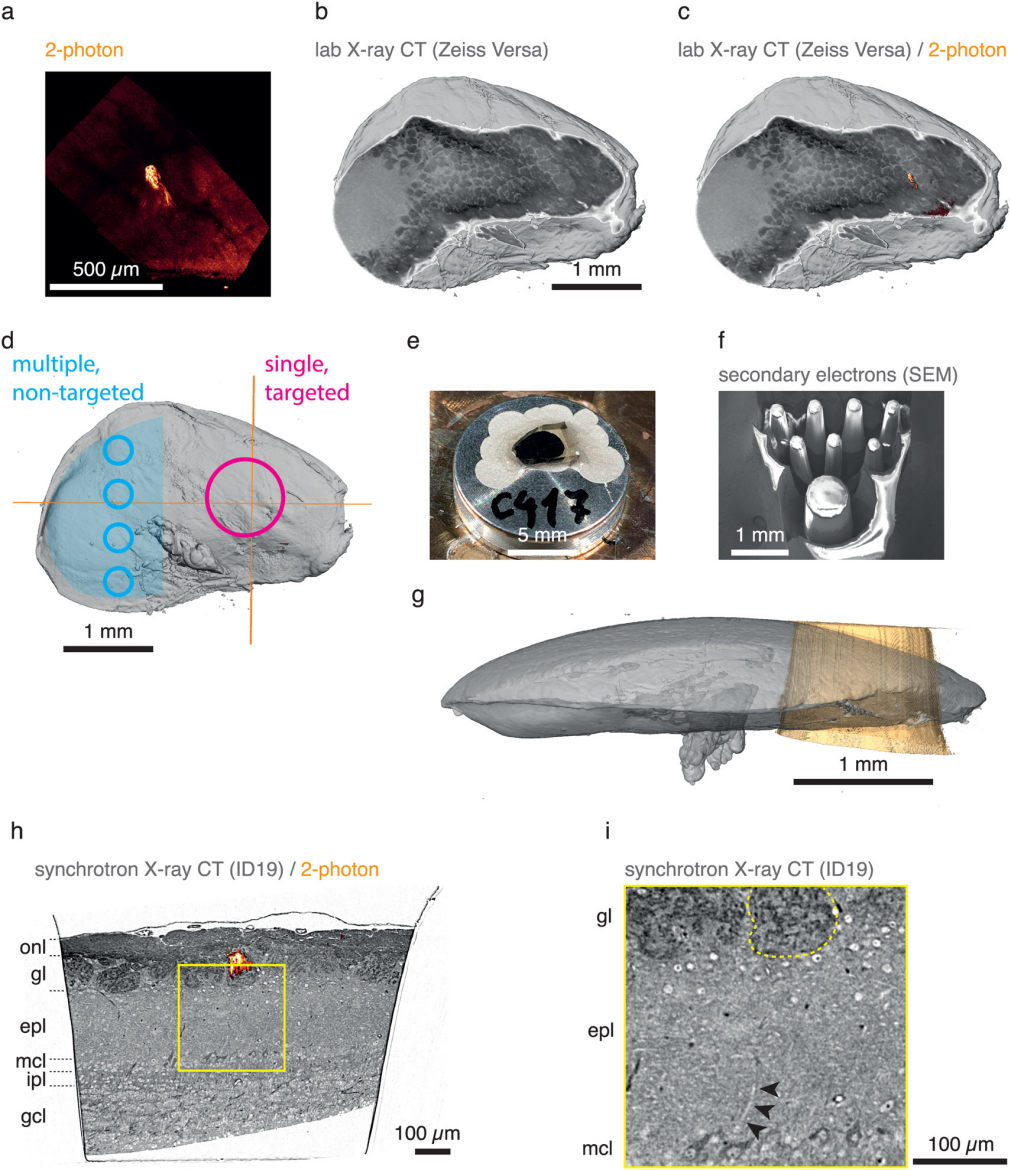
Preparation of targeted cylindrical sample for synchrotron x-ray imaging. (a–c) Mouse brain olfactory bulb tissue with a fluorescently labeled MOR174/9 glomerulus was imaged *ex vivo* with two-photon microscopy, stained, embedded, and imaged with lab x-ray CT (LXRT). Both two-photon (a) and LXRT (b) datasets were warped to the same space making it possible to identify the MOR174/9 glomerulus in the stained and embedded sample (c). (d) A targeted region of interest (magenta circle) was defined so it would contain the MOR174/9 glomerulus (crosshairs) and their associated projection neurons. Other non-targeted regions of interest could be also programmed in the remaining specimen (blue circles). (e) and (f) The sample was then mounted on a standard SEM pin (e) and the excess tissue was milled with the fs laser, leaving the carved pillars (f). (g) The milled pillar (orange) was mounted individually and imaged with parallel-beam synchrotron x-ray computed tomography with propagation-based phase contrast (SXRT) at the ID19 beamline of the European Synchrotron Radiation Facility. This x-ray tomography technique provides near-*μ*m detail nondestructively in heavy metal-stained, epoxy resin-embedded samples of the biological soft tissue. The CMI approach allowed warping the 2p data to the LXRT (gray volume) and to this new SXRT dataset. This panel shows the actual position of the milled pillar (orange, segmented from SXRT data) within the original sample volumes before milling (gray, segmented from LXRT data). (h)–(i) Warping the 2p data onto the SXRT dataset revealed the position of the fluorescently labeled MOR174/9 glomerulus in the resin-embedded pillar. (i) As previously reported, the SXRT data can provide a subcellular biological context across the imaged landscapes, such as an apical dendrite of a mitral cell (black arrowheads) evolving in straight trajectory toward the genetically identified MOR174/9 glomerulus (circled with a dashed yellow line). *onl*, olfactory nerve layer; *gl*, glomerular layer; *epl*, external plexiform layer; *mcl*, mitral cell layer; *ipl*, internal plexiform layer; *gcl*, granule cell layer.

In multiscale and multimodal imaging, high-resolution subvolumes are embedded in a lower resolution context. This is achieved by first performing lower resolution imaging (light microscopy or, e.g., parallel beam synchrotron tomography) followed by targeted higher-resolution imaging (e.g., volume EM). Typically, a destructive sample preparation approach based on ablating or milling away the tissue is used to obtain a volume small enough for the high-resolution imaging techniques. This, however, implies that only one subvolume can be acquired at the highest resolution. For high-value specimens (e.g., those carrying lengthy functional imaging or behavioral experiments preceding anatomical investigation), it would be advantageous to acquire several neighboring subvolumes packed at the highest density, for example, to increase the number of sample replicates. Thus, we set to assess whether fs laser milling could be used to create several different targeted subvolumes from one single tissue specimen and at which packing density. To achieve this, we employed another fixed and stained olfactory bulb slice, mechanically trimmed to a ∼(1 mm)^3^ cube [Fig. 3(a)]. Using the combined fs-SEM system, we marked 16 locations for individual pillars of 500 *μ*m in height and 150 *μ*m in diameter [Fig. 3(b)]. To maximize the usable volume, we aimed to create pillars in close spatial proximity. Pilot experiments suggested that pillar walls could be milled as vertical as 6.0° ± 0.8° (n = 3 specimens, n′ = [13, 18, 16] pillars per specimen) [Figs. 4(a) and 4(b)]. Indeed, this allowed us to reliably ablate material between marked circular regions [Figs. 3(c) and 3(d)] and extract pillars of 500 *μ*m in height [Fig. 4(d)]. Notably, not only was the entire length of the pillars freely accessible [Fig. 3(e)] but also the round pillar profile was consistently smooth [Fig. 3(f)], important for many x-ray applications.^10^ After defining the target regions, the milling time to cut the complete set of pillars from the specimen was ∼15 min. The separation between two adjacent pillars is defined by two parameters: the slope of its side and its height [Fig. 4(a)]. From a pillar design perspective [Fig. 4(c)], the steepest slope achievable (gray line) will determine the maximum height of the pillars that can be milled at a giving packing density. Our 6° steep walls would, therefore, allow milling 500 *μ*m tall pillars as long as they are arranged leaving a separation of ∼105 *μ*m between their edges at the top surface. Improvements that might allow for steeper slopes in the milled pillars will enable a denser packing. Three scenarios can be imagined [Figs. 4(e)–4(g)] if aiming to extract multiple 500 *μ*m tall and 200 *μ*m wide pillars from a sample with a footprint of ∼1 mm^2^: a separation between adjacent pillars from 200, 100 or 50 *μ*m would enable extracting 6, 9, and 16 pillars, respectively.

**FIG. 3.**
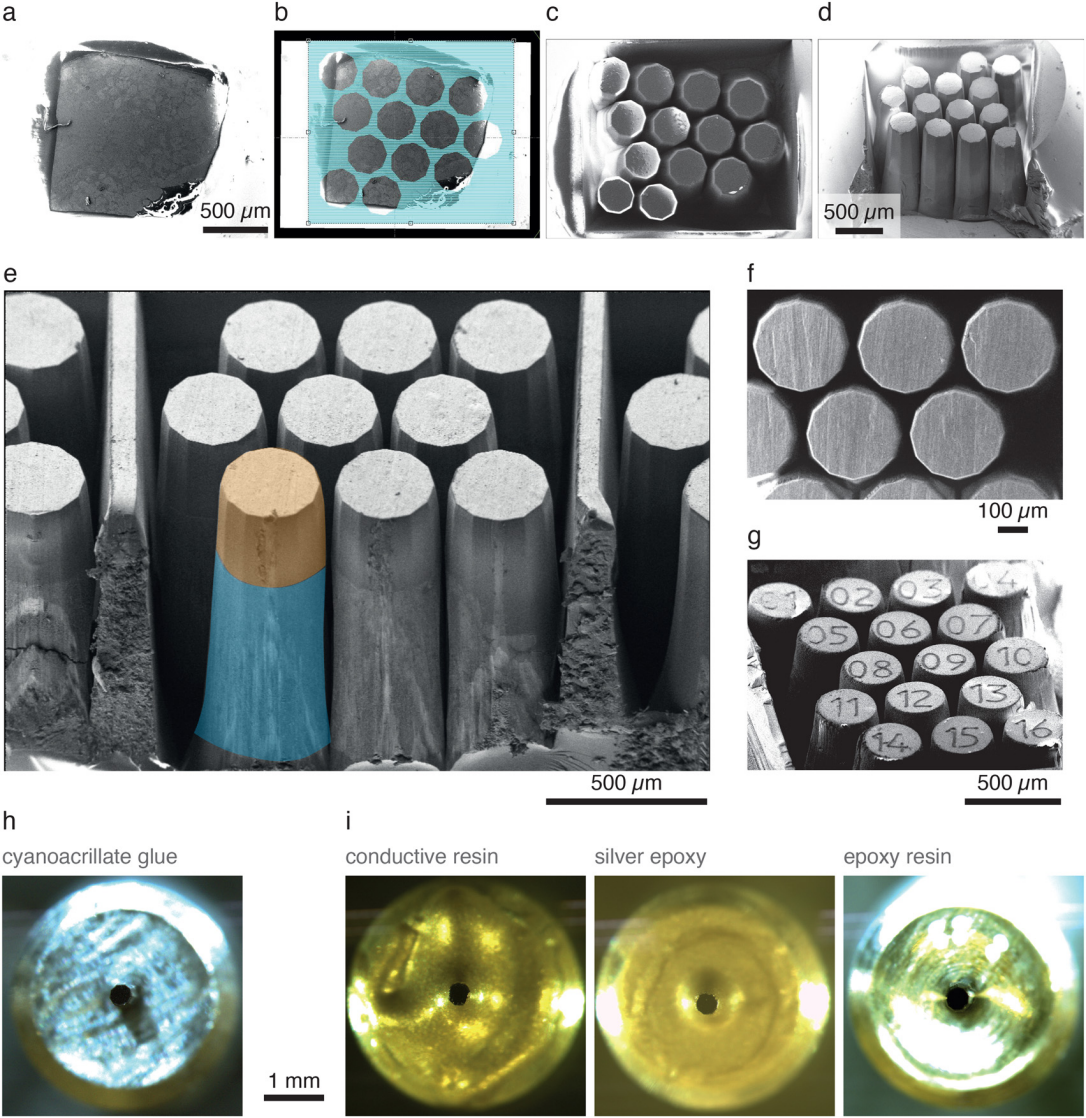
Production of pillar arrays. Multiple pillars can be extracted from a sample blockface of ∼1 mm^2^. The process involves imaging the blockface with scanning electron microscopy (a) and configuring the desired milling plan (b). The pillars milled present sharp edges and can, therefore, be densely packed (c)–(f). Finally, engraving of pillar IDs is possible following a similar procedure (g). (h)–(i) Pillars can then be separated and individually mounted (h) and embedded with appropriate materials to make them suitable for the intended follow-up imaging technique (i).

**FIG. 4.**
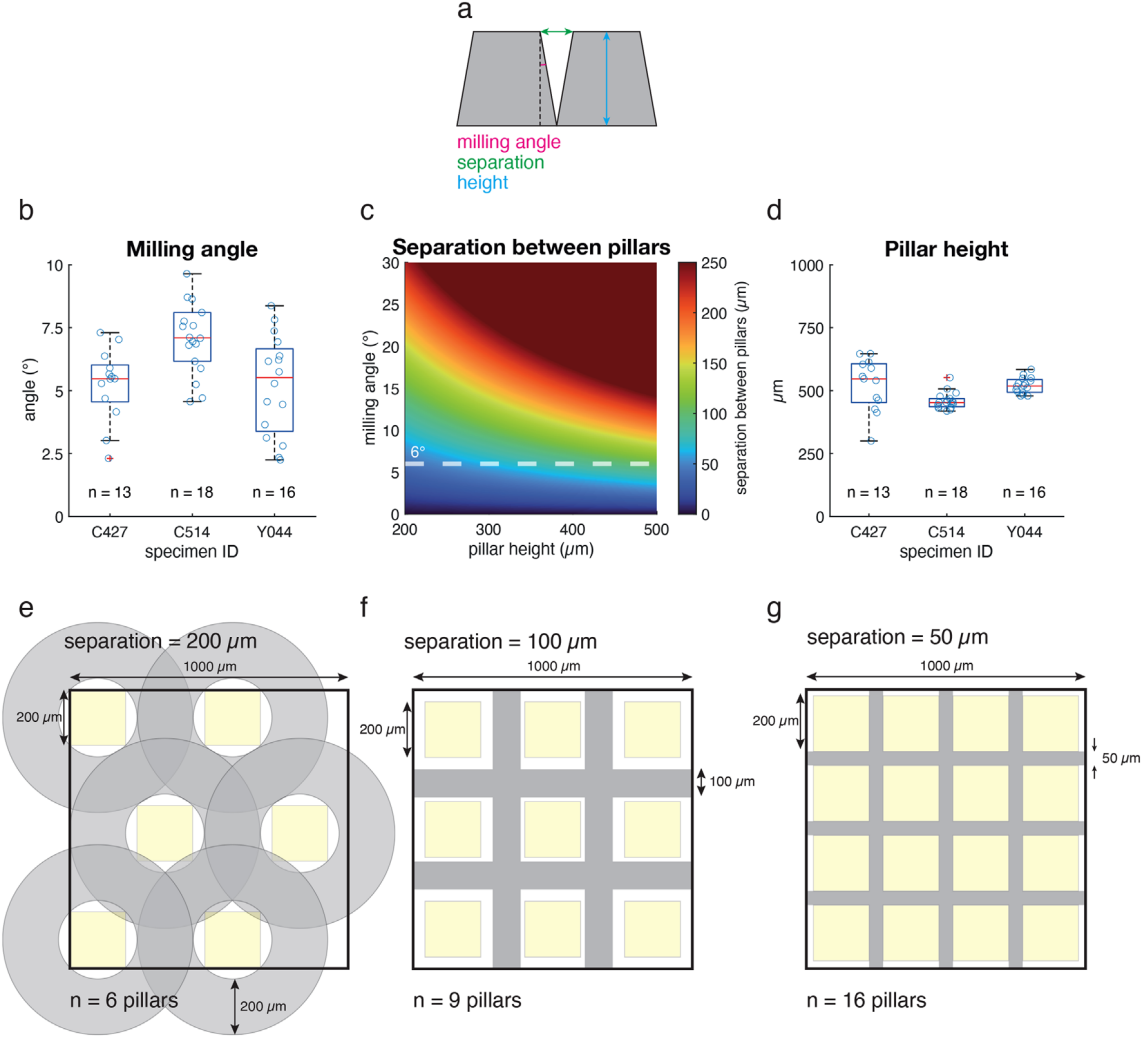
Multiple pillars can be extracted from neighboring locations within a 1 × 1 mm^2^ blockface. (a)–(d) The milling angle achieved in multiple neighboring samples enabled dense packing of pillar footprints, leaving <200 *μ*m between the blockfaces at the sample surface, yet still consistently recovering pillars 500 *μ*m tall. (e)–(g) Further improvements in the steepness of the milling process might allow for even tighter region of interest (ROI) packing density in a 1 mm^2^ blockface.

In a final step, pillars need to be separated from their common base to allow for individual re-mounting. To simplify the identification of individual pillars, we decided to “engrave” numbers to each individual pillar using the fs laser, using the same parameter settings as for the pillar milling, but directing the laser beam over the pillar top in a single pass only to create numbers with a depth of approximately 5 *μ*m [Fig. 3(g)]. This made it possible to mechanically separate all pillars simultaneously and store the pillars in individual holders for future use [Figs. 3(h) and 3(i)]. Furthermore, we could eventually control the milled depth from the laser power and number of hatchings employed, resulting in a range from <25 to >300 *μ*m in depth (Fig. S2 of the supplementary material).

Thus, as the laser beam can be guided on any arbitrary trajectory on the sample surface, it, indeed, allows users to cut out any shape from a specimen and to optimize the sample geometry to the needs of the final imaging or sectioning device. It is, therefore, possible to prepare several samples from the initial specimen, thus increasing the experiment's efficiency and allowing for the combined analysis of multiple spatially disperse regions in the same specimen.

Multiscale and multimodal imaging is a very powerful approach to link the different properties and length scales typical of biological tissues. However, sample preparation presents a critical challenge. Here, we introduce the fs laser as a powerful tool that complements mechanical trimming approaches. Using different brain tissue samples, we demonstrate that a fs laser allows for reliable targeting of subregions of different sizes. This is particularly pertinent when small volumes need to be extracted and separated individually for downstream imaging (e.g., for lengthy higher-resolution volume EM acquisitions) and which might require <100 *μ*m-precise targeting. Fs lasers can be programmed to specific sample positions using coordinates from upstream volume LXRT or SXRT imaging (mirroring recent approaches combining LXRT with automated ultramicrotome milling,^17^ albeit at significantly higher packing density, Fig. 4). Importantly, sample geometry can be essentially freely chosen, making it possible to create, e.g., cylindrical shapes optimized for tomography approaches.

There are several milling techniques that can be used for creating small specimens, like the pillars required in this work for synchrotron x-ray computed tomography. Ga^+^ liquid metal ion source (LMIS) FIBs have been the *de facto* method for milling microscale specimens, such as transmission electron microscopy (TEM) lamellae and micropillars. Ga^+^ LMIS are excellent for precision milling but have a low typical material removal rate at ∼2 × 10^1^ *μ*m^3^ s^−1^.^31^ The relatively recent introduction of Xe+ inductively coupled plasma (ICP) FIB has increased maximum material removal rates, at the cost of probe size, to typical rates of ∼7 × 10^2^ *μ*m^3^ s^−1^.^31^ However, even these gains are not sufficient to mill the cubic millimeters of material required to create the pillars required in a reasonable timeframe. Femtosecond laser ablation allows milling rates up to and beyond 3 × 10^5^ *μ*m^3^ s^−1^, enabling cubic millimeters of the material to be milled in times below 1 h.^31^ Using a femtosecond pulse width has been shown to produce minimal thermal damage to sample surfaces. Surface damage is typically in the order of 0.5 *μ*m thick for material systems, such as silicon, in the form of laser induced periodic surface structures.^1^ The fast milling rate of the fs laser along with the minimal surface damage caused by the laser pulses made this technique ideal for milling these macroscopic pillars, which required large volumes of material removal while keeping damage zones small. We corroborated this when we imaged the pillars using FIB-SEM (Fig. 1): a ring of redeposition of material was visible around the edge of the pillar, extending 0.5–1 *μ*m from the outer surface, but its presence did not impact FIB milling performance. Milling was only affected by the roughness of the pillar's surface, enabling optimal milling performance in smooth pillars.

Unlike mechanical trimming that relies on a flat blade trimming 2D faces, laser ablation operates from one single direction, ablating tissue in a line. This makes it possible to extract multiple target sample regions from one tissue specimen, including regions that are separated by only ∼100 *μ*m (Fig. 3). Such a dense array of volumetric ROIs (in this case pillars) minimizes any loss of material during the cutting process and enables parallel downstream processing and high resolution imaging of samples from neighboring regions. While this packing density may not be sufficient to follow individual biological features across neighboring pillars on their own, these can be mapped to previously obtained SXRT datasets in a correlative multimodal imaging workflow, thereby enabling inter-pillar matching of biological features at the *μ*m scale (Fig. 2).

Overall, a fs laser makes it possible to reliably extract multiple samples at targeted neighboring locations from resin-embedded soft tissue specimens and represents a very useful tool for developing robust correlative multimodal imaging workflows in life sciences.

See the supplementary material for supplementary Figs. (S1 and S2) and materials and methods.

## Data Availability

The datasets presented in this study can be accessed through https://wklink.org/2389 (C417_LXRT), https://wklink.org/8293 (C417_SXRT) and https://wklink.org/2803 (C427_SBF-SEM). The data that supports the findings of this study are available in the repository https://github.com/cboschp/fsLaser_CMI. The landmarks and warping engine linking the LXRT and SXRT datasets are available in the repository https://github.com/FrancisCrickInstitute/warpAnnotations, Ref. 32.
